# Validation of a novel *Mho* microarray for a comprehensive characterisation of the *Mycoplasma hominis* action in HeLa cell infection

**DOI:** 10.1371/journal.pone.0181383

**Published:** 2017-07-28

**Authors:** Birgit Henrich, Freya Kretzmer, René Deenen, Karl Köhrer

**Affiliations:** 1 Institute of Medical Microbiology and Hospital Hygiene, Medical Faculty of Heinrich-Heine-University Duesseldorf, Duesseldorf, Germany; 2 Biological and Medical Research Centre (BMFZ), Medical Faculty of the Heinrich-Heine-University Duesseldorf, Duesseldorf, Germany; Miami University, UNITED STATES

## Abstract

*Mycoplasma hominis* is the second smallest facultative pathogen of the human urogenital tract. With less than 600 protein-encoding genes, it represents an ideal model organism for the study of host-pathogen interactions. For a comprehensive characterisation of the *M*. *hominis* action in infection a customized Mho microarray, which was based on two genome sequences (PG21 and LBD-4), was designed to analyze the dynamics of the mycoplasma transcriptome during infection and validated for *M*. *hominis* strain FBG. RNA preparation was evaluated and adapted to ensure the highest recovery of mycoplasmal mRNAs from *in vitro* HeLa cell infection assays. Following cRNA hybridization, the read-out strategy of the hybridization results was optimized and confirmed by RT-PCR. A statistically robust infection assay with *M*. *hominis* strain FBG enabled the identification of differentially regulated key effector molecules such as critical cytoadhesins (4 h post infection (pI)), invasins (48 h pI) and proteins associated with establishing chronic infection of the host (336 h pI). Of the 294 differentially regulated genes (>2-fold) 128 (43.5%) encoded hypothetical proteins, including lipoproteins that seem to play a central role as virulence factors at each stage of infection: P75 as a novel cytoadhesin candidate, which is also differentially upregulated in chronic infection; the MHO_2100 protein, a postulated invasin and the MHO_730-protein, a novel *ecto*-nuclease and domain of an ABC transporter, the function of which in chronic infection has still to be elucidated. Implementation of the *M*. *hominis* microarray strategy led to a comprehensive identification of to date unknown candidates for virulence factors at relevant stages of host cell infection.

## Introduction

*Mycoplasma hominis* is the second smallest, self-replicating mycoplasma species with 559 protein-encoding genes in type strain PG21, of which 220 are predicted to be *M*. *hominis*-specific [1]. This cell wall-less bacterium is found as a commensal in the urogenital tract of sexually active individuals but is also a potential pathogen leading to bacterial vaginosis, pelvic inflammatory disease, septic arthritis, preterm birth or even neonatal meningitis [2]. The patho-physiological mechanisms that enable this genetically heterogeneous commensal to become pathogenic are mostly unresolved. As attachment to host epithelial cells is thought to be the crucial step in infection, the identification of cytoadhesive membrane proteins in *M*. *hominis* strain FBG, such as the P80 secretin and the lipoproteins P50/Vaa, P60 and OppA [3–5], were the first propagated virulence factors of *M*. *hominis*. OppA, which ubiquitously functions as the substrate-binding domain of oligopeptide importers [6], additionally carries a unique *ecto*-ATPase activity in *M*. *hominis*. This *ecto*-ATPase was demonstrated to be essential for OppA-mediated cytoadhesion, induction of ATP release and apoptosis of *M*. *hominis* FBG-colonized host cells [7, 8].

The reduced number of protein-coding genes makes *M*. *hominis* an excellent model organism for studying host-pathogen interactions in detail. As *M*. *hominis* has the capacity to invade human host cells [9–11], an *in vitro* HeLa cell-based infection model system was established for the comprehensive characterisation of the host cell response to an *M*. *hominis* infection at different stages of infection [12]. Cytoadhesion of *M*. *hominis* strain FBG to the HeLa cell surface at 4 h post infection (pI) affected immune response and signal transduction pathways. Host cell genes involved in cell-cycle regulation, growth and death were highly differentially upregulated. Mycoplasma invasion, which started at 48 h pI led to the expression of lysosomal host cell genes involved with bacterial lysis, and in a chronically infected HeLa cell line (336 h pI) components of the ECM-receptor interaction pathway and phagosome-related integrins were markedly increased. The IL1B-dominated immune response affected MAPK signalling, cytokine-cytokine interactions and apoptosis. For the first time, these *in vitro* data show the complex, time-dependent reaction of the infected host cell directed towards mycoplasma clearance [12] and raises questions concerning the mycoplasma response.

Global approaches for the identification of molecules of *M*. *hominis* as participants in host-cell infection have rarely been described. Sequencing of virulent *M*. *hominis* strains associated with microbial burden in intra-amniotic infection and preterm birth has led to the detection of severe truncations in two surface-localised lmp proteins (Lmp1 and Lmp-like). In addition, a “gene of interest C” was identified and postulated as virulence factor due to its absence in less virulent strains such as PG21 [13]. Interestingly, the hypothetical, 55 kDa protein encoded by this gene, carried a signature of bacteriocin-processing endopeptidases that are known to be involved in growth inhibition of other bacterial species thus improving their own growth. In 2016, Goret et al. published a large-scale expression study of *M*. *hominis* lipoprotein genes upon contact with human dendritic cells (hDCs) [14].

The study presented here deals with the validation of a novel *Mho* microarray as a useful tool for the comprehensive characterisation of the transcriptomic action of *M*. *hominis* strain FBG at the different stages of an *in vitro* HeLa cell infection.

## Material and methods

### *Mho* microarray design

*Mycoplasma hominis* (Mho) custom microarrays were designed by OakLabs GmbH (Berlin, Germany). At the time of design (Nov. 2013), probe oligonucleotide sequences were selected from two publically available Mho genomes, the 0.67 Mbp ATCC 23114 genome [1] and a 0.72 Mbp draft genome sequence of ATCC 27545 (acc-no. ARQG00000000.1; https://www.ncbi.nlm.nih.gov/Traces/wgs/?val=ARQG01) using OakLabs´ proprietary design algorithm. This comprised of 15.208 partially replicated Mho oligonucleotides including a custom set of P50/VAA adhesin sequences and FBG genes and as well as Agilent standard control features (RNA spike ins, dark corner, bright corner, etc.). After microarray spotting (Agilent Technologies, Waldbronn, Germany) *M*. *hominis* FBG specific probe sequences were identified by hybridizing genomic FBG DNA and summarised in an FBG-specific mask file to extract strain specific hybridization signals from gene expression experiments. Out of 13,708 non-redundant Mho oligonucleotides in total, 1,668 displayed specificity for the *Mycoplasma hominis* strain FBG (Oaklabs, Berlin Germany), the provisional draft genome of which was recently calculated to be 0.75 Mbp. Microarray platform definition data was deposited at NCBI GEO (GPL23264).

### Cell culture and infection conditions

The human cervical carcinoma cell line HeLa S3 (uninfected or infected by FBG) was cultivated in DMEM and *M*. *hominis* FBG was cultivated in arginine-medium as described in detail previously [2]. In 75 cm^2^ cell culture flask, HeLa cells (10 million cells per flask) were infected with 50 MOI (multiplicity of infection) of FBG for 1 h, 4 h, 48 h and 336 h; at each time point the experiment was performed in duplicate. Total RNA was purified from each infection assay for microarray-based transcriptome analyses. RNA from time point 1h of infection was used as a reference. Recovery rates of *M*. *hominis* and HeLa cells at each time point of infection were estimated by TaqMan based quantification of HeLa (h*gap*) and *M*. *hominis* (*hit*A) genome equivalents [15], enabling the calculation of cell counts and the respective bacteria to host cell ratios.

### Nucleic acid preparations and gene expression analyses

Nucleic acids were prepared from each 75 cm^2^ cell culture flask after washing the adherent HeLa cells twice with 10 ml PBS and subsequent lysis of the washed cells in 650 μl RLT Buffer (RNeasy Kit; Qiagen GmbH, Hilden, Germany). 50 μl lysate was used for genomic DNA preparation and 600 μl lysate for total RNA preparation as previously published [12]. Before using the RNA as a template for RT-PCR, contaminating traces of DNA were digested with DNase I [6]. To show that genomic DNA had been sufficiently removed from the RNA samples the same amount of RNA (as the negative control) and cDNA were simultaneously subjected to p80 qPCR. RNA samples were judged as DNA-free when delta CT-values (cDNA–mRNA) were higher than 10; corresponding to less than 0.01% contaminating DNA. Total RNA integrity was checked using an Agilent 2100 Bioanalyzer system (Agilent Technologies, Waldbronn, Germany). All samples of this study showed high quality RNA integrity numbers (RIN 9.2–9.8). RNA was further analysed by photometric Nanodrop measurements (Thermo Fisher Scientific GmbH, Dreieich, Germany) and quantified by fluorometric Qubit RNA assays (Life Technologies).

Synthesis of cDNA and subsequent fluorescent labelling of cRNA was performed on duplicates of each experimental condition according to the manufacturer´s protocol (Agilent One-Color Microarray-Based Exon Analysis/Low Input Quick Amp WT Labelling Kit; Agilent Technologies, Waldbronn, Germany) with some modifications. Briefly, 100 ng of total RNA were converted to cDNA, followed by *in vitro* transcription and incorporation of Cy3-CTP into the nascent cRNA. To compensate for the prominent AT content of mycoplasma, equal volumes (0.24 μl) of Cy3-UTP (Enzo Life Sciences, Lörrach, Germany) were added to the labelling reactions. After fragmentation, the maximum amount of labelled cRNA (2.4 μg) was hybridized to Agilent *Mycoplasma hominis* GE 8x60k Microarrays (OakLabs GmbH, Berlin, Germany/Agilent Technologies, Boeblingen, Germany) for 48 h at 65°C and scanned as described in the manufacturer´s protocol.

Signal intensities on 20 bit tiff images were calculated by Feature Extraction (FE, Vers. 11.0.1.1; Agilent Technologies, Waldbronn, Germany) using a custom grid file (061652_D_F_20140129; OakLabs GmbH, Berlin, Germany). Data analyses were conducted with GeneSpring GX (Vers. 12.5; Agilent Technologies). Probe signal intensities were quantile normalised across all samples to reduce inter-array variability [16]. Input data pre-processing was concluded by baseline transformation to the median of all samples.

After grouping of replicates according to their respective experimental condition a given oligonucleotide had to be expressed above background (i.e. the probe fluorescence signal was detected within the 20th and 100th percentiles of the raw signal distribution of a given array) in both duplicates in any one of two or both conditions to be further analysed in pairwise comparisons of conditions (4 h infected vs. 1 h infected, 48 h infected vs. 1 h infected, 336 h infected vs. 1 h infected). Differential gene expression was statistically assessed using moderated T-tests. Data are available under GEO accession number GSE97596.

### Pathway analyses

Primary pathway analysis was performed online using the Kyoto Encyclopedia of Genes and Genomes (KEGG) website (http://www.genome.jp/kegg/pathway.html; vers. 7/21/2011). Entrez Gene IDs (including aliases) of differentially expressed genes were searched against *M*. *hominis* ATCC 23114 pathways within the KEGG basic pathway mapping tool. HTML output was extracted and integrated into a local database for detailed enrichment analyses of differentially regulated genes.

### RT-qPCR

Oligonucleotides were designed using Probefinder (Roche Applied Science) (https://qpcr.probefinder.com). Primers are listed in Table 1.

**Table 1:** Primers used.

**MHO-Gene**	**FBG_RTqPCR Primer**	**Sequence (5‘-3‘)**
0300	0300_F	CCTACAATTATGGTTCACGGTATTTT
0300	0300_R	ACGAATACCTCCAGCAGTTGA
0530	0530_F	CAATTGCTACAACAGCGCTTA
0530	0530_R	TTCTTTTTGCGATTGATTTGC
1510	1510_F	CAGAACACTTCTATATGCAGCAGTAA
1510	1510_R	CTGGAACTTTTGCAAGTCAGTG
1540	1540_F	GGGGGTATGAAACAAAGAATTG
1540	1540_R	AATGCTGTTGTTGGTTCATCAG
2080	2080_F	TGAAATTAGGCAAGATAATAGGACAA
2080	2080_R	CGGCAACACAACAGGTAAAAT
2100	2100_F	TTTGAAACAACCGCTAAGAAATTA
2100	2100_R	CTGCAACTGCTTCTGCCTTA
3470	3470_F	TGCCGAAGCAACCAAATCA
3470	3470_R	CTGCTGCTGAAAGTTTAGAAATAATTG
3610	3610_F	TTCAAACAAAAACAAGGGCTTT
3610	3610_R	TTTTCTTAGCGGATCATTGTCA
**MHO-Gene**	**FBG-RT-Primer**	**Sequence (5‘-3‘)**
0710	710_F1	GCAATAATTGTTTTAAGCCTATCG
0710	710_F2	GCATTATTAGTCTTGGCTTCCT
0720	720_F1	GACTTTCTGCTGCTTGTGGT
0720	720_F2	TAAGAAACAAGTCCAAGGTCAAG
0730	730_F1	GTCAGTAGGATCCAAGACAGTGAATAA
0730	730_F2	CAAGCAATTGGAGTGAAAAAACATATGATAGAT
0740	740_F	TGTTGGTATAAGAGGCGAATCTC
0740	740_R	TTCAACTAATTTTATGCTTCCTTCAA
0750	750_R	ATAGCATGATACTCATACCAGAGTTTG
0760	760_R	GTAAATTATAAACATTGGGAGAATTGC
0770	770_R	ATGAACTAAATTTGCAATACTACATTC

RNA (1 μg) was converted to random-primed cDNA in a total volume of 40 μl according to the instructions of the manufacturer (Invitrogen, Life Technologies, Darmstadt, Germany), followed by threefold dilution in 10 mM Tris/HCl, pH 7.5. The qPCR assays were then carried out in a total volume of 25 μl consisting of 1 × MesaGreen MasterMix, 5 mM MgCl_2_, Amperase, 300 nM of each primer and 2.5 μl of the cDNA solution, which was derived from 20 ng RNA. Thermal cycling conditions were as follows: 1 cycle at 50°C for 10 min, 1 cycle at 95°C for 10 min followed by 45 cycles of 95°C for 15 s and 60°C for 1 min for amplification, and 1 cycle at 95°C for 15 s, 1 cycle at 60°C for 1 min. The product was than heated from 65°C to 95°C with an increment of 0.5°C/15 s and the plate read for melt curve analysis to check the identity of the amplicon. Each sample was analysed in duplicate. Cycling, fluorescent data collection and analysis were carried out in an iCycler from BioRad Laboratories (Munich, Germany) according to the manufacturer's instructions. Relative quantification of transcripts based on the ΔΔCt-method with respect to the housekeeping gene *gap* [17] or the differentially unregulated *lgt* gene of the Mho-microarray approach, and time point 1h pI [18].

### RT-PCR

RNA was prepared from exponential phase cultures of *M*. *hominis* strain FBG and cDNA synthesised as described previously [19]. Overlapping regions of the MHO genes 720–770 were amplified using the Long Range PCR Kit (Qiagen, Hilden, Germany) by standard PCR conditions (initial cycle of 3 min at 93°C; 35 cycles of 15 s at 93°C, 30 s at 50°C, 10 min at 68°C). PCR products were separated on 0.8% agarose gels and stained with ethidium bromide. Primers used are listed in Table 1.

## Results

### Establishment of a robust cRNA synthesis workflow

To be able to sensitively analyse mycoplasma gene expression profiles and to evaluate the changes in transcript abundance over time in complex mixtures of human host and Mho transcripts, two RNA sample preparation strategies were tested based on 1) human rRNA depleted samples, and 2) total RNA analysis. The rRNA depletion approach required a comparatively large amount of total RNA input (5–10 μg), and the procedure in our hands turned out to be quite variable regarding the amount and quality of the depleted samples (assessed by Qubit and Bioanalyzer analyses. Additionally, the eluted RNA samples were not compatible with the following Agilent Low Input Quick Amp WT Labelling protocol without introducing substantial modifications (volume and concentration of the input material). Finally, after evaluating possible suitable modifications the labelling reactions again showed varying efficiencies, partially not reaching the Agilent cRNA QC specifications, and hybridization of technical replicates consequently showed poor correlation of probe signal intensities.

Alternatively, we optimized the standard Agilent cRNA synthesis and labelling workflow using total RNA preparations as the starting material. Therefore, the standard amount of 100 ng of total RNA was used, supplementing the IVT labelling reaction with an additional volume of Cy3 labelled dUTP to compensate for the pronounced AT content of the *Mycoplasma hominis* genome. This reproducible procedure resulted in high quality cRNA within the Agilent labelling efficiency specifications. To account for the possible underrepresentation of Mho transcripts within the complex host-pathogen samples, the amount of cRNA for Mho microarray hybridisations was increased four-fold. Finally, hybridization time was extended to 48 h as compared to the standard 17 h. The combination of these modifications resulted in a robust and reproducible, cost-effective cRNA labelling and hybridization workflow. HeLa cRNA was shown not to have an impact on mycoplasma cRNA hybridization.

### Differentially expressed mycoplasma genes over time in the HeLa infection model

**Fig 1:**
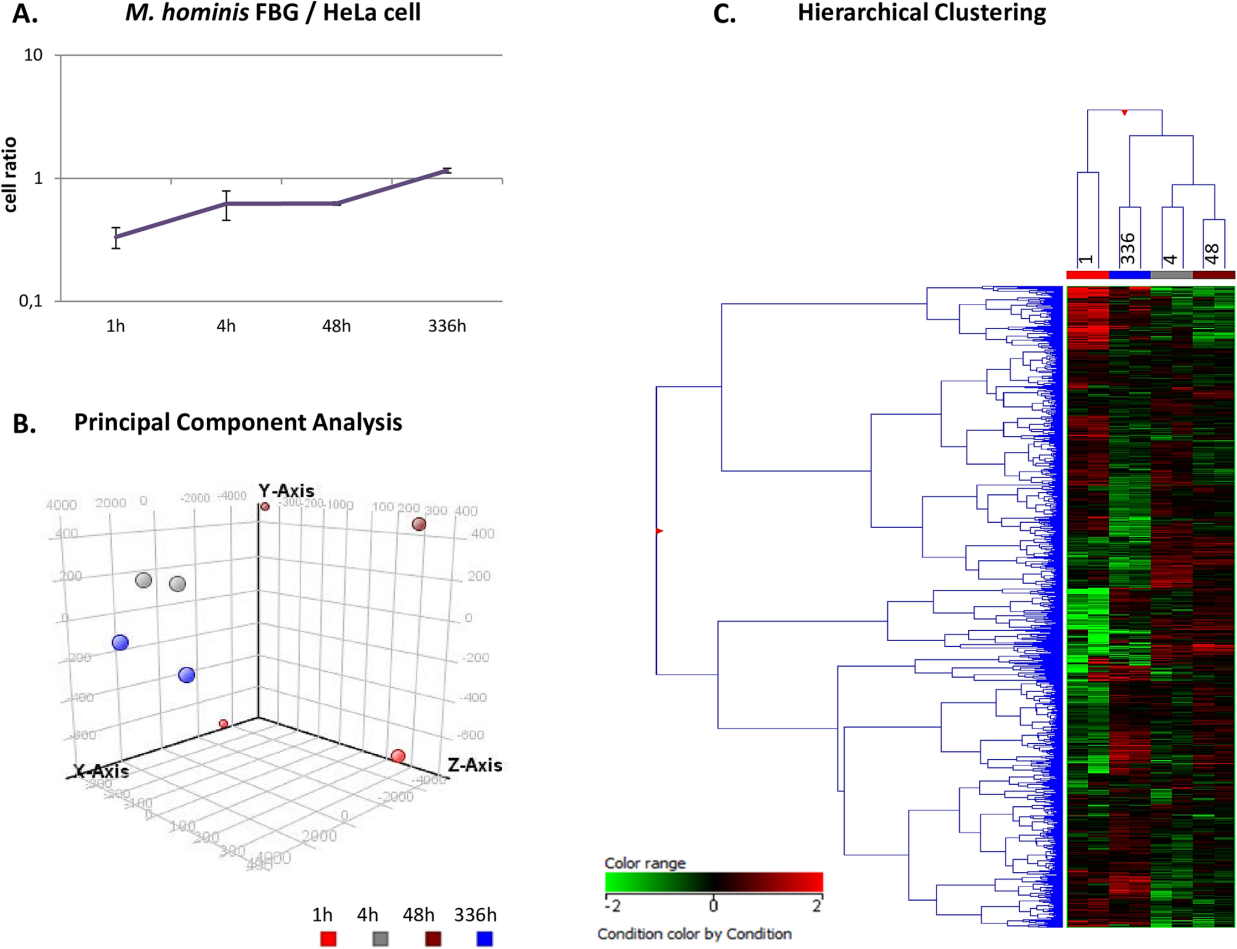
Clustering of differentially regulated *M*. *hominis* FBG genes at each time point of HeLa cell infection. A, ratio of cytoadhesive *M*. *hominis* cells to HeLa cells (MOI 50) and at each time point of infection (1 h, 4 h, 48 h and 336 h); B, principal component analysis; C, hierarchical clustering of the differentially regulated FBG genes at each time point, measured in biological duplicates.

**Fig 2:**
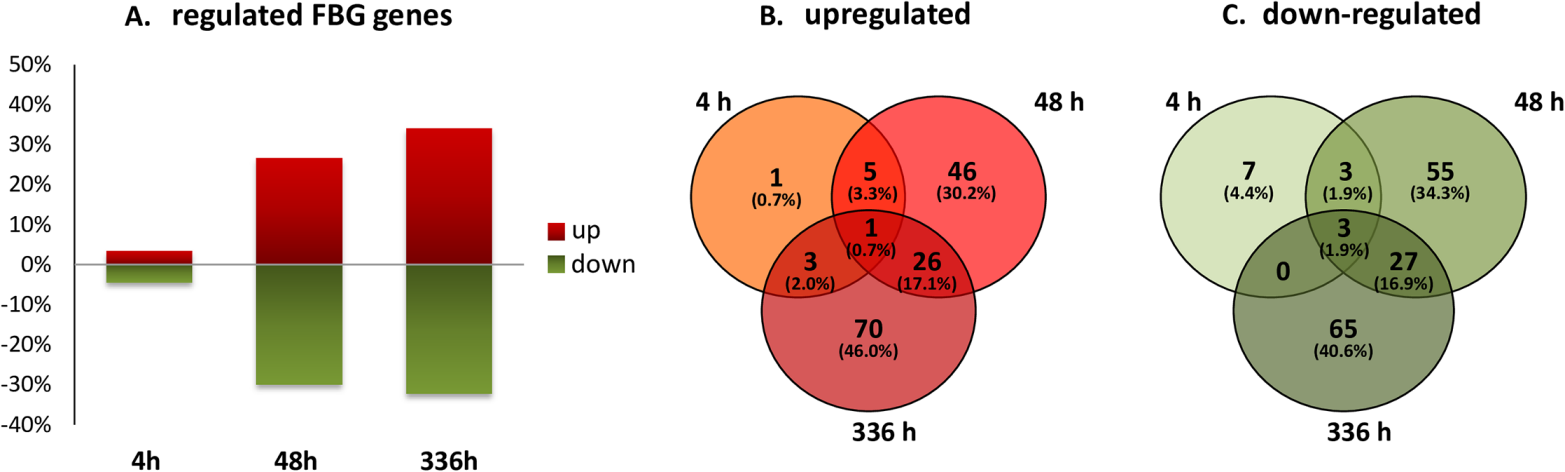
Distribution of differentially regulated *M*. *hominis* FBG genes. Numbers of differentially up- and downregulated (> 2-fold) genes of *M*. *hominis* strain FBG at 4 h, 48 h and 336 h post infection compared to time point 1 h. A, numbers of differentially up- and downregulated genes; B, Venn diagram of differentially upregulated genes; C, Venn diagram of differentially downregulated genes.

To elucidate the pathogen’s action at the different stages of *in vitro* HeLa cell infection, four post infection time points (pI) were chosen for microarray gene expression analyses: 1 h pI to monitor the baseline of transcription, 4 h pI to examine mycoplasma action at host cell attachment, 48 h pI to capture transcriptional changes at the initiation of invasion and 336 h pI to examine mycoplasma’s action during chronic infection. Each time point was analysed in duplicate. Starting with a 50-fold multiplicity of infection, the ratio of attached and/or invasive mycoplasma to HeLa cells continuously increased from 0.3-fold (1 h) to 1.1-fold (336 h) (Fig 1A). Both, the number of HeLa cells and mycoplasma cells increased over the time, although the rate of increase was greater for *M*. *hominis*. Total RNA was prepared from these infection assays. The mycoplasma transcriptome changes were determined by comparative Mho microarray analyses based on the FBG-specific mask file. Principle component analysis (Fig 1B) illustrates the hierarchical clustering of the biological duplicates of each time point of infection (Fig 1C). 294 of the 296 differentially regulated FBG genes were differentially expressed by more than 2-fold at least at one time point of infection. Of these, 3.4% (n = 10) were up- and 4.4% (n = 13) were downregulated at 4 h post infection. At 48 h post infection the number of up- (26.5%; 78/294) to down- (29.9%; 88/294) regulated genes increased and was highest at 336 h pI: 100 genes (34.0%) upregulated and 95 (32.3%) downregulated (Fig 2). Remarkably, 128 (43.5%) of the differentially regulated genes in the *M*. *hominis* genomes of PG21 and LBD4 encode hypothetical proteins of unknown function. In type strain PG21, the percentage of differentially regulated genes encoding hypothetical proteins (42.8%) was higher than the percentage of all annotated genes in the genome encoding hypothetical proteins (37.4%). Thus, especially mycoplasma-specific proteins seem to be regulated in infection and may represent a reservoir of proteins putatively involved in all bacterial-host interactions. This hypothesis was corroborated by the finding that more than half of these differentially regulated *M*. *hominis* genes of unknown function (59%) were also found in other mycoplasma species, such as *M*. *arginini*, *M*. *arthritidis* and *M*. *canadense* (S1 Table; regulated hypothetical genes). A list of all differentially regulated genes is shown in S1 Table.

### *M*. *hominis* pathways affected in HeLa cell infection

**Fig 3:**
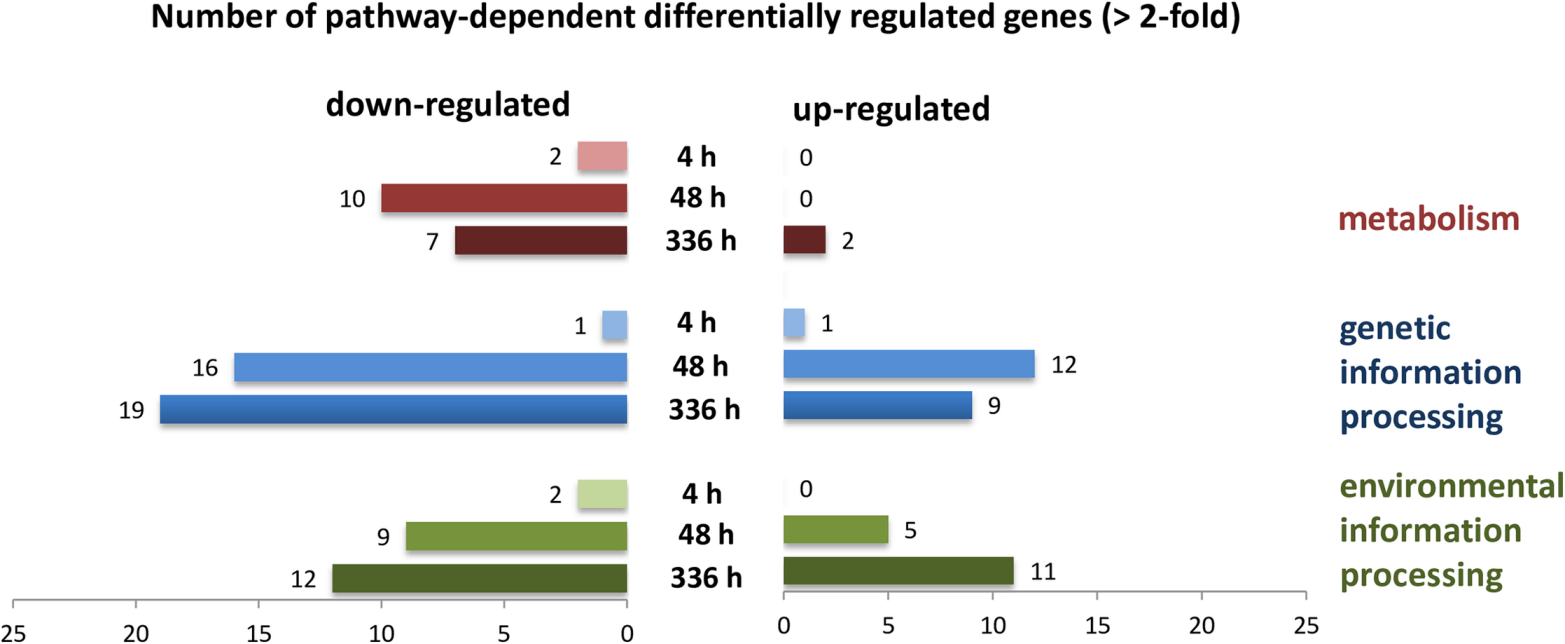
Pathway-dependent transcript regulation. Differentially regulated *M*. *hominis* FBG genes at 4 h, 48 h and 336 h pI were sorted to KEGG-pathway maps and integrated into those belonging to the Metabolism, Genetic or Environmental Information Processing and Cellular Processes. The number of differentially up- and downregulated pathway genes >2 fold are shown.

To look at a functional clustering of differentially regulated genes with known or predicted functions, we mapped the differentially expressed transcripts of the microarray analyses onto KEGG pathway maps of *M*. *hominis* ATCC 23114 (type strain PG21) [20]. KEGG pathway maps represent knowledge of molecular interactions and reaction network systems for *Metabolism*, *Genetic Information Processing*, *Environmental Information Processing* and *Cellular processes*. Then we calculated the ratio of differentially regulated genes per total number of genes in each pathway (S2 Table). Nearly all pathways were affected most at 48 h post infection (Fig 3) suggesting the highest mycoplasma activity during infection at the stage of colonisation and initiation of HeLa cell invasion. However, the ratio of differentially regulated mycoplasma genes remained constantly high between 48 h and 336 h pI in metabolic pathways of other amino acids and transcription; representing pathways with only a few pathway members found in *M*. *hominis*. The percentage of pathway members differentially regulated in 1.) *Folding*, *Sorting and Degradation of Genetic Information Processing*, 2.) *Bacterial Secretion System of Environmental Information Processing* and 3.) *Quorum Sensing of Cellular Processes* continuously increased during the time of infection indicating highest action of these pathway members in the “well-established” chronic infection.

### The most differentially regulated genes at each time point after infection

In order to obtain a more detailed understanding of the mycoplasma transcriptome changes we next focused on the highest differentially up- and downregulated genes at each time point of infection (Table 2).

**Table 2:** Highest differentially up- and downregulated *M*. *hominis* strain FBG genes at different stages of HeLa cell infection.

**NC_013511**	**4 h**	**Gene**	**Oligos**	**Description**
MHO_0150	**3.08**		1	hypothetical protein
MHO_0680	**2.47**		3	hypothetical protein
MHO_4710	**2.47**	vapD	1	Virulence-associated protein D
MHO_5350	**2.34**		1	hypothetical protein
MHO_5170	**2.13**		1	hypothetical protein
MHO_0330	**2.10**	ktrB	3	Potassium uptake protein KtrB
MHO_3390	**2.07**		1	hypothetical protein
MHO_4950	**2.05**		2	hypothetical protein (ABC permease)
MHO_3720	**2.03**	p75	2	P75 protein precursor
MHO_1010	**2.01**	rpsT	2	30S ribosomal protein S20
MHO_3470	**1.99**	vaa	2	Vaa surface lipoprotein adhesin
MHO_0720	***-2*.*48***		2	hypothetical lipoprotein
MHO_0740	***-2*.*41***		3	ABC transporter ATP-binding protein
MHO_0760	***-2*.*24***		3	ABC transporter permease protein
MHO_0730	***-2*.*20***		3	SNase-like lipoprotein
MHO_0750	***-2*.*12***		3	ABC transport permease protein
MHO_1080	***-2*.*26***		1	ATP-binding protein
MHO_5120	***-2*.*22***	parC	3	Topoisomerase IV subunit A
MHO_4530	***-2*.*21***		1	hypothetical protein
MHO_3770	***-2*.*17***	licA	2	LicA-like protein
MHO_3320	***-2*.*10***	upp	3	uracil phosphoribosyltransferase (EC 2.4.2.9)
MHO_4110	***-2*.*09***		2	hypothetical protein
**NC_013511**	**48 h**	**Gene**	**Oligos**	**Description**
MHO_2100	**13.80**		2	hypothetical protein
MHO_0680	**4.20**		3	hypothetical protein
MHO_4740	**3.83**		1	hypothetical protein
MHO_1050	**3.82**	thrS	1	threonyl-tRNA synthetase
MHO_1390	**3.76**		2	hypothetical protein
MHO_2040	**3.67**		1	hypothetical protein
MHO_1400	**3.66**		3	hypothetical protein
MHO_4930	**3.49**		1	hypothetical protein
MHO_3300	**3.43**	rnhB	2	ribonuclease HII
MHO_3470	**3.41**	vaa	2	Vaa surface lipoprotein adhesin
F807_00050	**3.33**		2	MLBD4_01025; hypothetical protein
MHO_1860	***-5*.*95***	secD	3	Protein-export membrane protein
MHO_0050	***-4*.*55***	dnaN	3	DNA polymerase III subunit beta
F807_00530	***-4*.*54***		1	MLBD4_00395; hypothetical protein
MHO_3840	***-4*.*42***	ackA	2	Acetate kinase
MHO_4520	***-3*.*89***	pcrA	1	ATP-dependent helicase
MHO_0040	***-3*.*88***	dnaA	2	Chromosomal replication initiator protein
MHO_1870	***-3*.*87***	hisS	3	Histidyl-tRNA synthetase
MHO_1700	***-3*.*82***	recU	1	recombination protein U
MHO_1070	***-3*.*66***		1	hypothetical protein
MHO_2040	***-3*.*57***		1	hypothetical protein
MHO_2470	***-3*.*47***		3	hypothetical protein
**NC_013511**	**336 h**	**Gene**	**Oligos**	**Description**
F807_00222	**15.37**	int	1	MLBD4_02580, MhoV1 integrase
MHO_3260	**10.37**		1	hypothetical protein
MHO_4010	**8.49**	rpmG	1	50S ribosomal protein L33
F807_00584	**7.76**	*dcm*	1	MLBD4_01840; DNA methylase (cytosine-specific)
MHO_1960	**7.14**		1	hypothetical protein
MHO_4740	**7.06**		1	hypothetical protein
MHO_4440	**6.87**		1	hypothetical protein
MHO_1700	**6.72**	recU	1	putative recombination protein U
MHO_1330	**6.35**		1	hypothetical protein
F807_00503	**6.21**	*mod*	1	MLBD4_00085, DNA methylase (adenine-specific)
MHO_1440	**5.81**		1	hypothetical protein
MHO_3400	***-10*.*48***	trxB	1	thioredoxin reductase
MHO_0660	***-8*.*25***		1	hypothetical protein
MHO_1850	***-7*.*36***	ruvB	1	holliday junction DNA helicase ruvB
MHO_1940	***-6*.*95***	hpt	2	Hypoxanthine-guanine phosphoribosyltransferase
MHO_0350	***-6*.*25***	pepC	1	Aminopeptidase C
MHO_1500	***-6*.*05***	comEB	1	Deoxycytidylate deaminase
MHO_1170	***-5*.*88***	pheS	1	phenylalanyl-tRNA synthetase alpha subunit(pheS)
MHO_0860	***-5*.*82***	acpD	4	Acyl carrier protein phosphodiesterase
MHO_2350	***-5*.*71***	truB	1	tRNA pseudouridine synthase B
MHO_3880	***-5*.*63***	rpiB	2	ribose-5-phosphate isomerase

At 4 h post infection only ten genes were differentially upregulated more than 2-fold, including the virulence-associated protein D (*vap*D) gene, the potassium uptake protein (*ktr*B) gene and the p75 lipoprotein gene and six genes encoding hypothetical proteins, one of which is predicted to encode a multi-drug efflux pump (MHO_0150), one an RpoE-homologue (MHO_0680), which enables a rapid adaption to environmental changes [21], and one an ABC permease (MHO_4950). The *vaa* gene was shown to be upregulated by approximately 2-fold. Expression of a cluster of five genes was differentially downregulated by more than 2-fold (MHO_720—MHO_760). These genes are consecutively positioned on the same genome strand and separated by less than 87 nt suggesting a polycistronic organisation. They putatively encode an ABC transporter with two lipoprotein domains (MHO_720 and _730), an ATP-binding domain (MHO_740) and two permease domains (MHO_750 and _760). Interestingly, the lipoprotein MHO_730 putatively functions as a nuclease as it carries the PFAM motif PF00565 of the secreted thermostable nuclease SNase of *Staphylococcus aureus* [22].

At 48 h post infection the MHO_2100 gene was highly expressed by more than 13-fold compared to the start of infection (1 h). Ten genes were upregulated by 3-4-fold, all of the which encode hypothetical proteins except the two genes *thr*S and *rnh*B, which encode enzymes involved in translation and replication processes, and the variable adherence associated *vaa* gene. The 10 highest differentially downregulated genes at 48 h pI showed a -3.5 to -5.95-fold change and code for proteins involved in protein export (*sec*D) and genetic information processes such as translation (*his*S), repair and replication (*dna*N, *dna*A, *pcr*A, *rec*U).

In the chronic infection stage (336 h pI) recombination and repair processes were highly affected (*int*, +15-fold; *dcm*, +7.76-fold; *mod*, +6.21-fold; *rec*U, +6,72-fold; *ruv*B, -7.36-fold) and transcription of genes involved in translation (*phe*S and *tru*B) and metabolic pathways (*trx*B, *hpt* and *pep*C) were shut down. At this stage of infection expression of the postulated operon genes MHO_720–760 reached their maximum (+ 2.32-fold).

### Proof of differentially regulated mycoplasma genes over time during infection

**Fig 4:**
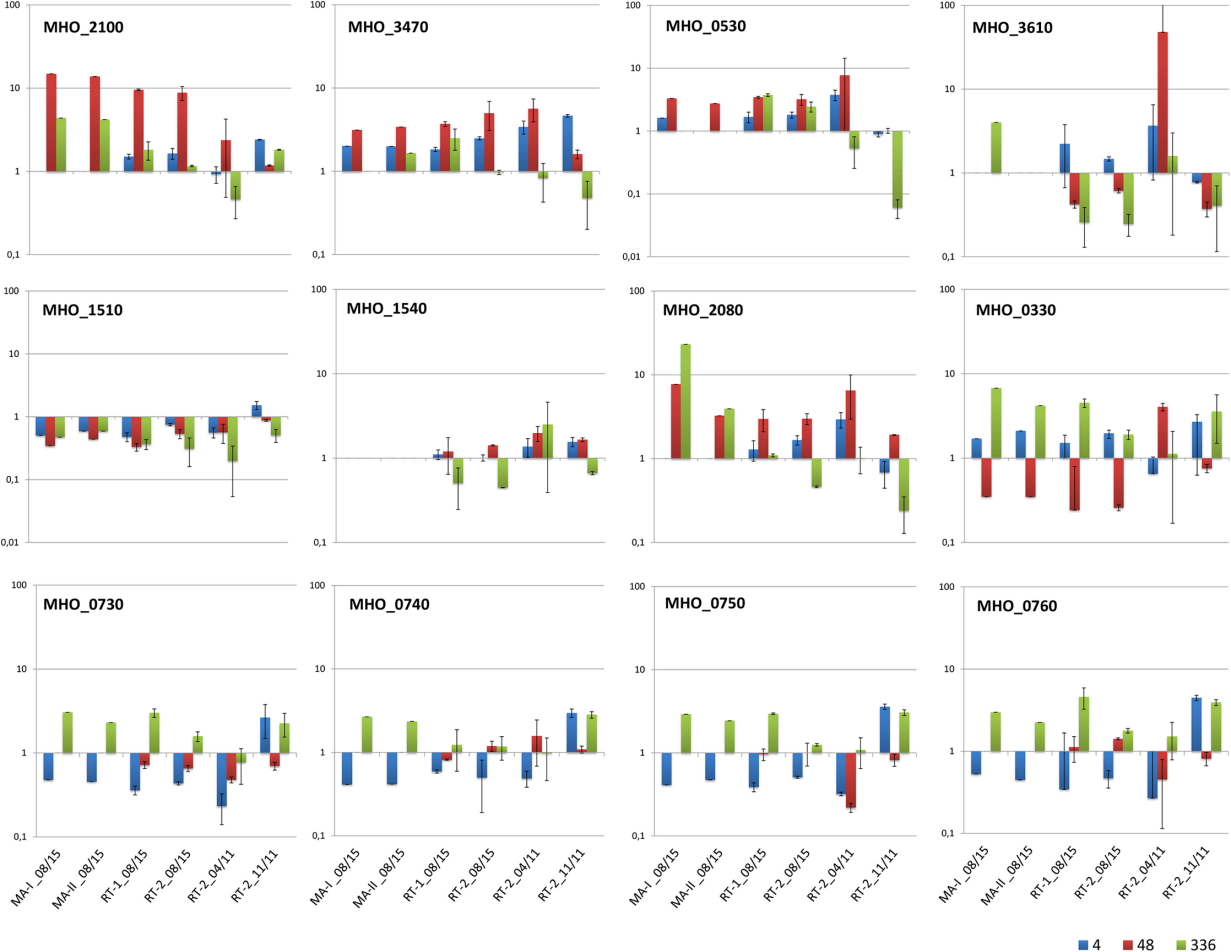
Comparison of microarray and RT-qPCR results. Total RNA of *M*. *hominis*-infected HeLa cells for 4 h, 48 h or 336 h was subjected to Mho-microarray or RT-qPCR analyses and the change in expression levels of the named genes, with respect to that at the start of infection (1 h for RNAs of 08/15 and 0 h for RNAs of 04/11 and 11/11), quantified as described in the Method section.

To verify the microarray results, transcript levels of selected genes were quantified by RT-qPCR using total RNA from the same infection assays as used for the Mho microarrays and, additionally, total RNA from two separate infection assays conducted before (Fig 4). These RNAs had formerly been used to characterise the host cell response to FBG infection [12]. Robustness of the microarray was demonstrated by technical replication of the array hybridisation using the same RNA samples (MA-I and MA_II 08/15). RT-qPCR results and microarray results correlated well for nearly all genes when using RNA from the same infection assay (-08/15). Comparable results were also achieved for most genes tested with RNA samples from an independent infection assay (04/11 or 11/11). Expression of lipoprotein MHO_2100 was greatly increased at an early stage of infection (4 h pI). Expression of MHO_3470 (Vaa) and MHO_0530 (lmp1) peaked at 48 h pI and transcription of the domains of the postulated ABC transporter, MHO_720–760 were greatest in chronic infection. Beside biological variances in infection, which were detected in one of the three independent infection assays, methodological differences between microarray and RT-qPCR results were less frequently observed (MHO_2080 and MHO_3610).

### Polycistronic organisation of MHO_730–760 genes in strain FBG

**Fig 5:**
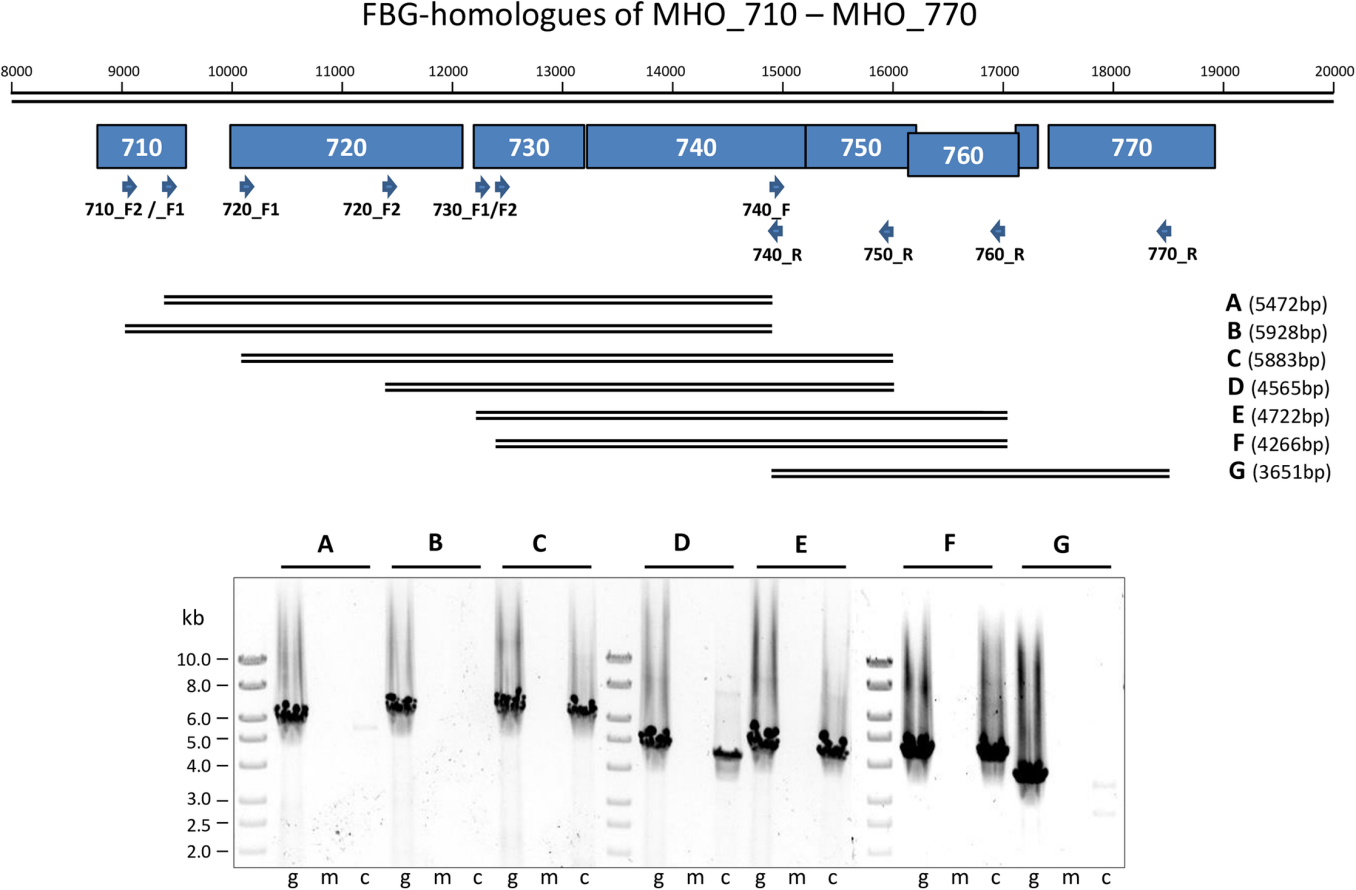
RT-PCR analysis of the postulated ABC transporter genes 730–760. The positions of the different amplicons are shown below the scheme of the MHO_710–770 gene region. The primers used (Table 1) and the lengths of the amplicons (A-G) are indicated. PCR products (A–G) for genomic DNA (g), mRNA (m) and cDNA (c) were separated on a 0.6% agarose gel and stained with ethidium bromide. M, Gene Ruler 1 kb DNA ladder (Fermentas).

Besides the identification of novel mycoplasma genes involved in host-pathogen interactions, the Mho microarray approach was also a useful tool for the detection of differentially co-regulated genes, which, when positioned side by side in the genome, suggests their polycistronic organisation. As transcript levels of the FBG homologues of MHO-730 to MHO_760 revealed the same induction from 4 h pI to 336 h pI, we examined the FBG genes of MHO_720 to MHO 770 for a common mRNA. RT-PCR products spanning the intergenic regions between MHO_720 and MHO_760 were obtained with primers hybridizing to MHO_720 and the 3'-end of MHO_750 (Fig 5B and 5C) and with primers hybridizing to the 5'-end of MHO_730 and to the 3'-end of MHO_760 (Fig 5E and 5F). No amplification occurred with primer pairs 710_F2 (or _F1) and MHO_740_R (Fig 5A and 5B) and 740_F and MHO_770_R (Fig 5G). These findings provided evidence that in strain FBG, MHO_730 is definitely polycistronically organized and co-expressed with the downstream genes MHO_740, _750 and _760.

## Discussion

To date, reports of an all-embracing characterisation of the pathogen activity in infection are rare [23–26]. This may be due to the magnitude of pathogens and the prerequisite of a customized microarray or the availability of other platforms such as RNA sequencing [27, 28], which was adapted to dual RNA sequencing of host and pathogen transcripts [29].

In this study we used a customized Mho microarray for the characterisation of the *M*. *hominis* action in *in vitro* HeLa cell infection. In several studies, enrichment of the pathogen transcripts from total RNA of the infection assay was used prior to labelling and microarray hybridisation [30–32]. This was generally undertaken by depletion of the host rRNA and/or mRNA. In this study, depletion of polyA-tagged HeLa mRNA or depletion of human rRNA was accompanied by loss of mycoplasma transcripts. This was probably caused by the presence of A-stretches in the AT enriched mycoplasma genome. Thus, total RNA was used for cRNA synthesis and microarray hybridization under the optimized conditions as reported above. With respect to the genetic heterogeneity of *M*. *hominis*, oligonucleotides of the Mho microarray were derived from two different genomes of *M*. *hominis*, PG21 and LBD-4, and other published gene sequences of *M*. *hominis* isolates. FBG specific oligonucleotides were then identified by hybridization of genomic FBG DNA to the Mho microarray. To date, eleven genome sequences of *M*. *hominis* have been published (https://www.ncbi.nlm.nih.gov/genome/), in which the annotated genes reveal the presence of mobile genetic elements, such as prophage MhoV-1 [33], insertion element ISMhom-1 [34] or *tet*M-carrying transposon [35]. MhoV-1 as part of the LBD-4 genome was the only one incorporated in the Mho microarray. Thus, the lack of information about mobile genetic elements in the FBG genome and potential absence of these genes restrict the Mho microarray-based transcriptome profiling to *M*. *hominis* type strain genes.

Using the advantages of a microarray, which offers a robust, reproducible hybridisation platform, we evaluated its performance by transcriptome analyses of *M*. *hominis* strain FBG in HeLa cell infection after 4 h, 48 h and 336 h. Time point 1h was selected as reference expression level of *M*. *hominis* strain FBG. Conditioning of the mycoplasma expression to the changed medium after 1 h was ascertained by identical transcript levels of some FBG genes (*gap*, p80 and *opp*A) in HeLa-cell-free FBG preparation, which remained constant from 0 h to 6 h. Nevertheless, it cannot be entirely excluded that the medium change can be responsible for transcriptome changes observed at later times for other metabolic genes (including lipoprotein genes involved in transport or transformation of nutrients). Of the 559 annotated genes, 296 genes were differentially expressed during the time course of infection. This is to our knowledge the first comprehensive *in vitro* analysis of the second smallest human pathogen in infection-stage dependent action on the host.

The functional KEGG analysis of differentially expressed genes was hindered by the fact that more than half of the genes encode hypothetical mycoplasma proteins of unknown function. In addition, publication-based knowledge of *M*. *hominis* was not really up to date in the KEGG pathway list. Although the function of OppA as the substrate binding domain of the oligopeptide permease of *M*. *hominis* has been known since 1999 [6] and *opp*A gene homologues are found for all mycoplasma *opp* operons [36], this information has not been embedded in the KEGG pathways and was added manually. In the quorum sensing pathway, which had not been described so far in mycoplasmas, the domains of the oligopeptide permeases Opp(A)BCDF and OppBC_2 putatively function as sensing proteins. Interestingly, Opp belongs to the family of ABC transporters and mycoplasmal ABC transporters were proposed by McAuliffe to function in bacterial communication systems such as quorum sensing [37].

Some other annotated domains of ABC transporters (MHO_3210; MHO_3520/MHO_3530; MHO_3610 to MHO_3630 and MHO_3820/MHO_3830) and the majority of lipoproteins (see below) are also not listed in *Membrane Transport System* and *Cellular Community*, respectively. As most of their genes were shown to be differentially regulated in time course of infection (see S1 Table) they should be registered in these KEGG categories.

### Lipoproteins as virulence factors

Due to the lack of a cell wall, surface exposed membrane proteins and lipoproteins are important key players for the initial interaction of mycoplasmas with their host. In *M*. *hyopneumoniae*, a swine pathogen, six out of 79 genes differentially expressed in lung infection encoded lipoproteins [32]; and in an *in vitro M*. *agalactiae* infection assay lipoproteins comprised one-fourth of the differentially regulated genes [38].

There are 43 predicted lipoproteins in the PG21 genome, the type strain of *M*. *hominis* [1], 84% of which have been identified at a protein level [14]. In an *in vitro* infection model 38 lipoprotein genes were differentially expressed after 4 and 24 h of co-incubation with human dendritic cells (hDC). A predominant overexpression at 4 h pI suggested an immediate action of surface localised mycoplasma proteins upon contact with hDCs. This is in good accordance to the findings in this study that the majority of these lipoproteins (n = 20/38 (52,6%)) were differentially expressed at 4 h and/or 48 h of FBG-HeLa infection and only 4/38 in long term infection. Remarkably, the same direction of gene regulation (up or down) between 4 h and 24 h (PG21) or 4 h and 48 h (FBG) was observed for 12 hypothetical lipoprotein genes and the well-known *lmp*3, *vaa*, p120 and p75 genes. Temporal expression of the well-known lipoprotein lmp1 differed between both infection models; it did not change within 48 h upon contact with hDCs [14] but increased in FBG upon HeLa cell contact from 4 h to 48 h pI.

Survival of *M*. *hominis* in the infected host relies on a balance between an intimate contact to the host cell for nutrition uptake and a strategy for immune evasion. Invasion of host cells by *M*. *hominis*, which could serve as a strategy to circumvent its demise, was demonstrated for several cell types. Invasion of HeLa cells was characterised in *in vitro* models [9, 12]. Invasion in spermatozoa was shown to lead to abnormal sperm morphology [10, 11]. In placental tissue *M*. *hominis* was found to be associated with intra-amniotic infection and preterm birth [13], and inside prostate cells it was shown to be associated with the development of prostate cancer [39]. In a computational study by Kahn et al. the *M*. *hominis* proteome of ATCC_27545 was screened for proteins with signatures that predict targeting of the host nucleus [40]. Their study was based on the hypothesis that intracellular pathogens modulate the normal function of the host cell by targeting their proteins in various organelles. Cell cycle and growth may thereby be affected in the nucleus, which results in tumor development [41]. 29 *M*. *hominis* proteins were computationally predicted to target the nucleus [40]. As hypothesized, most of the proteins (n = 16/29) function in processes affecting cell cycle and growth (translation, transcription, DNA replication and repair). In the transcriptome profile of FBG in HeLa infection more than half of these genes were expressed most at 48 h pI (n = 7), when mycoplasma invasion started, or at 336 h pI (n = 3), when invasion is well established in the chronically infected HeLa cell. Six proteins with nucleus-targeting signature have ATP-binding capacities; including MHO_740, which was demonstrated in this study to be part of an ABC transporter, expression of which was greatest in long term infection. The lipoproteins Vaa and Lmp1 were also predicted to target the host nucleus. Their expression was greatest at 48 h pI, when expression of lysosome-specific host proteins was increased promoting bacterial lysis. These data suggests a patho-physiological function of these lipoproteins in infection beside the mediation of a tight contact to the host cell (Vaa). Interestingly, virulent *M*. *hominis* isolates from women with preterm birth all carried severely truncated Lmp1 proteins [13]. Lmp1 truncation led to the formation of large mycoplasmal auto-aggregates [42]. Thus, it remains to be elucidated in future, whether such isolates can circumvent a lysosomal attack of the host due to the Lmp1-truncation and/or survive the host defence by protection of the interiorly positioned mycoplasmas in aggregates.

### Novel candidates for virulence factors

The Mho microarray-based transcriptome profiling of *M*. *hominis* FBG in HeLa infection has led to the identification of novel candidates for virulence factors, such as cytoadhesins (P75 (= MHO_3100)), invasins (MHO_2100) and transport systems acting at the initial phase of colonization (MHO_0150 with homology to Na+-driven multidrug efflux pumps; ABC permease MHO_0490) and transporters promoting mycoplasma survival in chronic infection (MHO_720–760).

We would like to shed more light on two 37 kDa lipoproteins as novel virulence factors; MHO_2100 and MHO_0730. MHO_2100 protein expression, which had already been demonstrated in type strain PG21 [14], was proven in strain FBG. The expected 37 kDa protein was immune-stained in FBG lysate using a polyclonal MHO-2100 antiserum demonstrating its abundance (S1 Fig). As MHO_2100 represented the highest differentially upregulated transcript at 48 h pI (+13.8 fold) its function as an invasion factor was postulated. This assumption was supported by the finding that the protein sequence carries a conserved sequence motif, TIGR01612 (AA118-307), which is common in the 235 kDa-family of reticulocyte binding/rhoptry proteins. These surface localised proteins are well-characterised in *Plasmodium* spp. as essential molecules for the process of parasitic invasion of reticulocytes [43]. Interestingly, MHO_2100 does not count among the vitally important genes of *M*. *hominis*. It is absent in the genomes of the pathogenic *M*. *hominis* strains Sprott [35] and SP2565 [44], but is present in the virulent strains AF1, AF3 and PL5 [13]. Thus its impact on mycoplasma invasion and type and severity of disease is still to be elucidated.

Contrarily, MHO_730 was detected in several mycoplasma species and identified as a nuclease-encoding gene. Activity of the membrane associated nucleases was shown to depend on divalent cations (Ca^2+^ in *M*. *bovis*, *M*. *genitalium*, *M*. *gallisepticum* and *M*. *hyopneumoniae;* and Mg^2+^ in *M*. *agalactiae* [45–49]). In *M*. *bovis*, it was shown to function as cytotoxic, secreted nuclease with the potential to induce apoptosis in macrophages [46]. In *M*. *hominis* strain FBG immunostaining revealed that it is not abundantly expressed and only detectable in FBG cultures at the early logarithmic growth phase (S1 Fig). A poly-cistronic organisation of MHO_730 with genes encoding domains of an ABC transporter was postulated for 11 mycoplasma species (incl. *M*. *hominis*) due to the adjacent position of their genes [45] and experimental evidence is provided by the present study. MHO_720 is positioned upstream in the operon of 10 mycoplasma species, encoding a further lipoprotein [45, 49]. The functional role and relation of this lipoprotein, the ABC transporter and the postulated nuclease are yet unknown and remain to be characterised in chronic infection, during which the transcription was most differentially upregulated.

### Host-pathogen interaction partners

Based on the comprehensive characterisation of the *M*. *hominis* action on HeLa cells at the different stages of infection presented in this study and the findings of the former characterisation of the host response to temporal FBG infection [12], a first *M*. *hominis*—host cell interplay in infection is proposed as follows:

The first contact of *M*. *hominis* to its host cell is mediated by cytoadhesins. The variable adherence associated P50/VAA protein of *M*. *hominis* is characterised by binding to sulphated host cell structures [4],which are enriched in the endometrium [50]. Vaa knockout mutants of *M*. *hominis* have been shown to be less adhesive [51], thus demonstrating Vaas’ cytoadherence function, but also indicating that additional surface molecules are involved in mycoplasmal attachment to the host, such as P60/P80, the multifunctional lipoprotein OppA and the novel cytoadhesin candidate P75 (MHO_3100). OppA was demonstrated to bind to HeLa cell surfaces by its ATPase moiety, and to extracellular matrix (ECM) molecules by the CS1 region, which is conserved in bacterial OppA proteins [52], thus enabling a modulation in host cell attachment. In this initial colonization stage the host cell *hsp*70 is down-regulated and the host reacts by upregulation of pro- and anti-apoptotic cytokines and ECM molecules. To date, a downregulation of *hsp*70 has been characterised only in *Helicobacter pylori* infection, where it leads to an inhibition of NO-induced apoptosis, mucosal damage and chronic infection [53]. This is in good accordance to the less severe, but more chronic infections associated with *M*. *hominis*. Upregulation of the virulence associated protein D (vapD), which has been characterised as bacterial ribonuclease [54] with an increased expression in biofilms [55], may also affect degradation of host mRNA in FBG infection. In *Haemophilus influenzae* vapD-derived mRNA degradation was demonstrated to be beneficial for bacterial survival [56]; probably in an biofilm-embedded and in an intracellular status. Increased expression of host ECM molecules increases the amount of mycoplasmal target structures thus facilitating endocytic uptake of *M*. *hominis*. *M*. *hominis* invasion is accompanied by down-regulation of its P75 and a variant expression of the host actin genes leading to a rearrangement of the actin cytoskeleton. This has been demonstrated in *M*. *hominis*-infected and *M*. *agalactiae*-infected Hela cells [12, 57]. Various bacterial toxins and effectors are known to target and modify the actin cytoskeleton thus disturbing the epithelial barrier functions and adherence of cells to the extracellular matrix [58]. In this scenario, the novel invasion candidate MHO-2100 may play a comparable role as effector of *M*. *hominis*. The initially strong (re)actions of host and pathogen attenuate in the chronic infection stage (336 h pI) including down-regulation of MHO_2100. Growth of the *M*. *hominis* infected HeLa cell, which was diminished in the first days of infection, synchronize to growth of an uninfected HeLa cell line, which suggests life in a fool's paradise. Transcription of distinct lipoproteins (Lmp3, MHO_2080) and cytoadhesins (P75, P60) increased suggesting a specialised function in chronic infection, at which the majority of mycoplasma cells colonize the surface of the HeLa cells [12]. However, phagocytic processes are ongoing and promoted by an increased expression of host cell serpins, bradykinin receptors and S100 proteins, while intracellular *M*. *hominis* cells reside in lysosomes and in exocytoplasmic protrusions of the HeLa cell [12]. Whether the differentially upregulated nuclease MHO_730 or the entire ABC transporter MHO_720–760 balance these host reactions, are involved in lysosomal escape or facilitate *M*. *hominis* exocytosis, remains to be elucidated.

## Conclusions

Implementation of a customized Mho microarray enabled the comprehensive characterisation of the temporal *M*. *hominis* transcriptome changes in HeLa cell infection and the identification of novel, formerly unknown candidate virulence factors at different stages of infection. This now enables further functional and mechanistic studies to confirm their involvement in the patho-physiology of infection.

## Supporting information

S1 Table Microarray dataset: Differentially regulated *M*. *hominis* genes [>1.3 fold] at 4 h, 48 h or 336 h HeLa cell infection. (XLSX)

S2 Table Number of differentially regulated, pathway-assigned *M*. *hominis* FBG genes. (XLSX)

S1 Fig Immunostaining of MHO_0730- and MHO_2100-homologues proteins in lysates of FBG-cultures and FBG-HeLa infection assays using polyclonal antisera. Protein lysates, which derived from A.: FBG-cultures at early (4) logarithmic growth to stationary growth phase (1), or B.: FBG-HeLa infection assays were separated on 12% SDS-PAGE. In Western blotting distinct proteins were immunostained by monoclonal antibodies (mAb) DC10 antiOppA), BA10 (antiP50/P42 of VAA), NB12 (antiP80), CG4 (antiP60) or polyclonal antisera (PAS) against MHO_2100 or MHO_730.
(TIF)
