# Supplementary figures and images for: Validation of a novel Mho microarray for a comprehensive characterisation of the Mycoplasma hominis action in HeLa cell infection
Source: PLoS One. 2017 Jul 28;12(7):e0181383. doi: 10.1371/journal.pone.0181383 (PMC5533444; doi:10.1371/journal.pone.0181383)

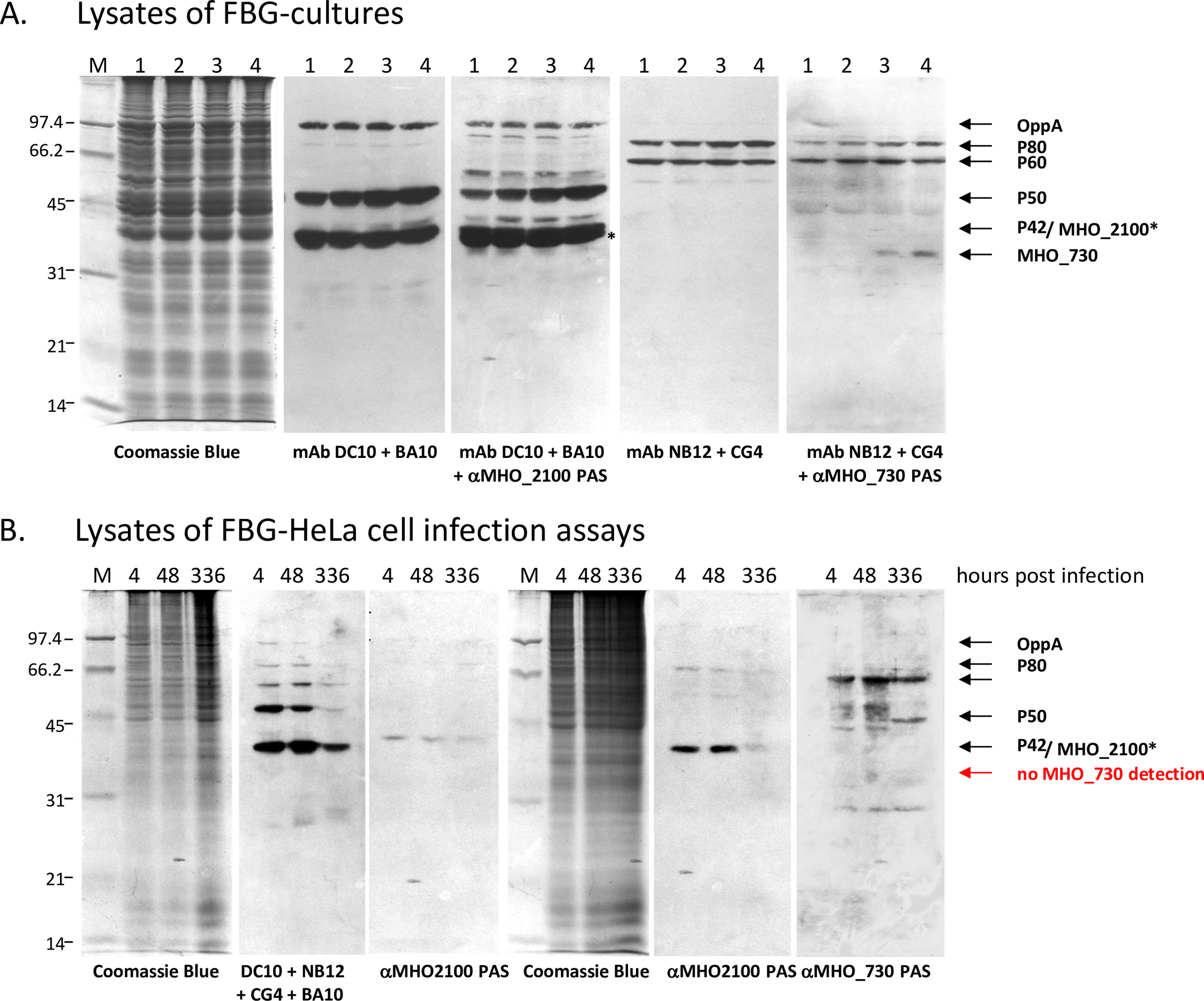

Supplement: S1 Fig — Protein lysates, which derived from A.: FBG-cultures at early (4) logarithmic growth to stationary growth phase (1), or B.: FBG-HeLa infection assays were separated on 12% SDS-PAGE. In Western blotting distinct proteins were immunostained by monoclonal antibodies (mAb) DC10 antiOppA), BA10 (antiP50/P42 of VAA), NB12 (antiP80), CG4 (antiP60) or polyclonal antisera (PAS) against MHO_2100 or MHO_730. (TIF) [file pone.0181383.s003.tif]
